# Evaluation of the Efficacy of Ultrasound-Guided Dry Needling Therapy and Exercise in Piriformis Muscle Syndrome

**DOI:** 10.7759/cureus.43804

**Published:** 2023-08-20

**Authors:** Derya Guner, Zeynep A Ozcete

**Affiliations:** 1 Pain Medicine Department, Izmir Health Sciences University, Tepecik Training and Research Hospital, İzmir, TUR; 2 Physical Medicine and Rehabilitation Department, Izmir Health Sciences University, Tepecik Training and Research Hospital, İzmir, TUR

**Keywords:** interventional ultrasonography, physical therapy modalities, pain measurement, neuralgia, exercise, ultrasound, dry needling, pain, piriformis muscle syndrome

## Abstract

Background: Piriformis muscle syndrome (PMS) is characterized by symptoms of buttock pain and numbness radiating to the back of the thigh due to irritation of the sciatic nerve. This study aimed to evaluate the efficacy of dry needling (DN) therapy and exercise programs on pain, neuropathic pain, physical function, and disability in patients with PMS.

Methods: Forty-four patients with PMS were included in the study. Patients were divided into two groups, those who were treated with DN three times once per week under ultrasound guidance and those who had an exercise program for three weeks. To identify the outcomes of the treatment modalities, pre-treatment and post-treatment first-month and third-month visual analog scale (VAS), Oswestry Disability Index (ODI) questionnaire, Lower Extremity Functional Scale (LEFS), and Douleur Neuropathique 4 (DN4) questionnaire scores were used.

Results: There was no statistically significant difference between the groups in baseline scores of VAS (p = 0.548), DN4 (p = 0.446), and LEFS (p = 0.880), but in the DN group, baseline ODI scores were significantly higher than in the exercise group (p = 0.001). The group comparisons showed no statistically significant differences in decreasing pain, reducing disability, and increasing functional status scores among the groups at post-treatment first-month and third-month assessments (p > 0.05).

Conclusion: Both treatment modalities are beneficial in reducing pain and disability, and increasing the functional status of the patients with PMS in three months of follow-up. In patients who cannot adapt to exercise programs, DN treatment under ultrasound guidance should be kept in mind as a minimally invasive treatment modality with no adverse effects.

## Introduction

Piriformis muscle syndrome (PMS) is a neuromuscular disorder that can cause symptoms of hip joint motion limitation, buttock pain and tenderness, and numbness radiating to the back of the thigh due to compression or irritation of the sciatic nerve [1,2]. In the etiology of PMS, myofascial trigger points are the most common cause, and also hypertrophy in the piriformis muscle tissue, inflammation, trauma, and anatomic variations of the piriformis muscle or sciatic nerve may cause the development of PMS [3]. Prolonged activities that irritate the piriformis muscle (including cross-legged sitting, long walking or sitting on a hard surface, running, or cycling) may exacerbate PMS symptoms [4]. Physical examination supports the diagnosis of PMS and may help to eliminate competing diagnoses. Multiple physical examination maneuvers (active piriformis test, Beatty test, flexion-adduction-internal rotation (FAIR) test, and Pace test) have been identified to help diagnose PMS but no physical examination maneuver is diagnostic in itself. Compression and deep palpation may also exacerbate buttock or gluteal pain [5]. Electrodiagnostic tests are usually normal in FMS and useful in excluding other conditions, such as lumbosacral radiculopathy [6]. There is no gold standard treatment option for PMS, and conservative treatment and lifestyle changes remain the mainstays for the treatment. Conservative approaches, including oral medications (non-steroidal anti-inflammatory drugs (NSAIDs), muscle relaxants, and neuropathic agents) and physical therapy modalities and exercise, aim to reduce local pain, muscle tenderness, and spasms [5].

Piriformis muscle stretching is an exercise technique in physiotherapy that is generally used for patients with PMS. Exercises focused on relaxing the piriformis muscle aim to increase the resting length of the muscle and reduce the potential sciatic nerve compression because of this tightness [7]. Local anesthetics, corticosteroids, botulinum toxin injections, dry needling (DN), acupuncture, manual overpressure, and massage treatments can be listed as other treatment modalities for PMS [8-10]. DN therapy is a treatment method where myofascial trigger points are stimulated using acupuncture needles or injection needles [11,12]. DN can be performed according to the anatomic landmark method or under ultrasound (US) guidance and fluoroscopic imaging. US guidance is important in the management of PMS, allowing imaging of specific deep muscle groups and avoiding complications such as procedural pain and damage to neurovascular structures [13]. There are no randomized controlled studies other than case series on the use and frequency of application of the US-guided DN techniques in PMS.

This study aimed to compare the effectiveness of DN treatment performed on the piriformis muscle once per week for a total of three times under US guidance and a three-week exercise treatment program in PMS. The secondary outcomes of this study were to evaluate and compare the effect of these treatment modalities on visual analog scale (VAS) scores, the Oswestry Disability Index (ODI), Lower Extremity Functional Scale (LEFS), and Douleur Neuropathique 4 (DN4) questionnaire scores in patients with PMS at three months follow-up.

## Materials and methods

In this prospective observational study, between January 2022 and May 2023, 175 patients with unilateral hip pain who presented to the pain and physical medicine and rehabilitation outpatient clinic were evaluated. Forty-four patients aged 18-70 years, who were clinically diagnosed as having PMS after excluding other hip, lumbar, sacroiliac region, and lower extremity pain etiology diagnoses were enrolled in the study. The diagnosis was based on the patient’s history and physical examination, bearing positive flexion, adduction, and internal rotation (FAIR) test, and/or tenderness or trigger point at the piriformis muscle area and/or revealing pain with maneuvers such as Freiberg’s maneuver (forceful internal rotation of the extended thigh in the supine position), Beatty’s maneuver (actively abducting the affected thigh in the lateral decubitus position), and Pace’s maneuver (resisted abduction of both thighs in the seated position). The demographic data (age, sex, occupation, and body mass index (BMI) scores) and the side of the pain were noted. Each patient in the study received and signed an informed consent form.

Despite all patients receiving oral medical treatment, their pain continued. The patients were divided into two groups, those who did accept DN treatment (group 1, n = 22) and those who received physical exercise treatment (group 2, n = 22). All patients in group 1 had DN treatment performed on the piriformis muscle once per week, a total of three times under US guidance. All patients in group 2 underwent an exercise program for three weeks.

Overall pain severity was measured using a 0 to 100 -mm scale VAS. Pain disability was assessed using the ODI questionnaire, and physical function was rated using LEFS. The presence and severity of neuropathic pain were evaluated according to the DN4. To identify the outcomes of the treatment modalities, pre-treatment and post-treatment first-month and third-month scores were used. LEFS consists of 20 items that rate the ability of functional status in performing different physical activities due to musculoskeletal problems affecting the lower extremities. Each question has a five-point scale, scored from 0 to 4 (0: unable to do the activity, 1: quite difficult, 2: moderate difficulty, 3: somewhat difficult, and 4: no difficulty). The total score ranges from 0 to 80, with higher scores indicating better functional status [14].

The Turkish validity and reliability study of LEFS was performed by Citaker et al. [15]. The DN4 questionnaire was developed by the French Neuropathic Pain Group in 2005 [16]. The Turkish validity and reliability study of the DN4 questionnaire was performed in 2010 [17]. The ODI questionnaire, which measures pain disability, consists of 10 questions, each with six options [18]. The questions include pain intensity, ability to work, perform self-care, sit, stand, travel, lift, sleep pattern, socializing, and sexual life. Every question is scored between 0 and 5 points. The highest score is 50 if all questions are answered, and indicates severe disability for the participant. The Turkish validity and reliability study of the ODI was conducted by Yakut et al. in 2004 [19]. Patients with comorbidities that prevented interventional treatment (e.g., inflammatory diseases, uncontrolled diabetes mellitus, uncontrolled hypertension, malignancy, pregnancy, and severe psychiatric disorders), patients with other diagnoses that might cause hip and leg pain (e.g., lumbar disc herniation, sciatic nerve injury history, and sacroiliac and hip joint pathologies), those aged under 18 years, and patients who did not accept interventional or physical therapy modalities were excluded from the study.

Exercise protocol

A home-based static self-stretching technique was described and patients were given an additional exercise plan of bilateral bridging, side leg raises with hip and knee flexion to 45 degrees and feet together by the therapist, and they were taught to refrain from sacral sitting, change their posture every 30 minutes, and lift heavy objects sparingly. The stretching position was sustained for 30 seconds, with 10 repetitions/sets enforced twice daily. A form containing exercise images was given to facilitate patient recall.

DN intervention protocol

**Figure 1:**
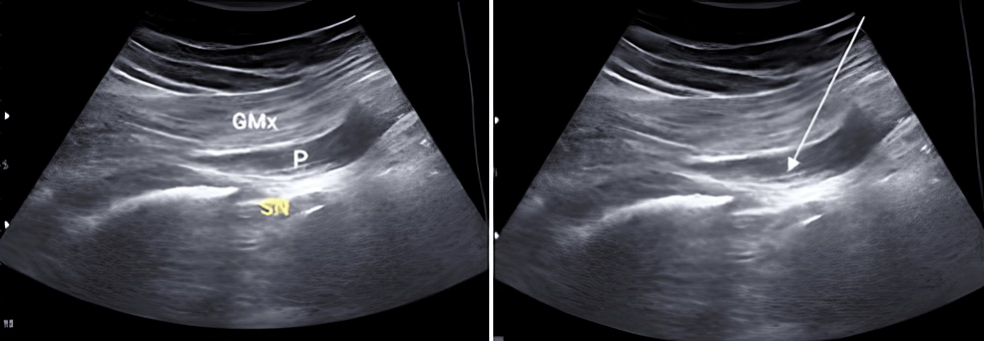
Ultrasound-guided piriformis muscle dry needling with in-plane technique from medial to lateral GMx: gluteus maximus muscle; P: piriformis muscle; SN: sciatic nerve; arrow: block needle.

After a signed informed consent form was obtained from each patient who underwent DN in the study, a total of three sessions of DN treatment were performed once per week by the same experienced pain physician (researcher) to the piriformis muscle under US guidance on all patients in the DN group. DN was performed in an intervention room under sterile conditions with the patient in the prone position. A pillow was placed under the abdomen to exclude lumbar lordosis. A low-frequency (2-5 MHz) transducer was placed in the gluteal region in a slightly oblique position and sonographic scanning was started. First, the large mountain shadow of the iliac crest and gluteus maximus muscle was sonographically visualized in the midline. Then, the transducer was shifted inferiorly until the sciatic notch was visualized. When the sciatic notch, sciatic nerve, and pear-shaped piriformis muscle above it were visualized, the patient's knee was flexed and the leg was rotated internally and externally, and the typical sliding motion of the piriformis muscle was observed. After the target point was determined, a 22-G spinal needle was inserted from lateral to medial or from medial to lateral using the in-plane technique (Figure 1) [20]. The needle was inserted and withdrawn several times until the switch response in the piriformis muscle was observed and extinguished.

Declarations

Ethical approval for the study was obtained from the Ethics Committee of the University of Health Sciences Turkey, İzmir Tepecik Education and Research Hospital (Decision No.: 2022/2/1; Date: 19.01.2022). The study was conducted in accordance with the Declaration of Helsinki. The patients were informed about the study and each provided oral and written informed consent.

Statistical methods

Normally distributed continuous data are presented as mean and standard deviation and non-normally distributed data are presented as median and interquartile range. Categorical variables were analyzed using frequency tables, and descriptive statistics were calculated for continuous variables. Independent sample t-tests and Mann-Whitney U tests were conducted to compare parametric and nonparametric continuous data, respectively. Pearson’s chi-square test was used to analyze categorical data in terms of groups. Non-parametric repeated measures comparisons for each group were analyzed using the Friedman test. In all hypothesis tests, p < 0.05 was considered significant. Odds ratios at 95% confidence intervals (CI) for each parameter were calculated for each variable. The statistical analysis was conducted using the IBM SPSS version 25.0 statistics package (IBM Corp., Armonk, NY).

## Results

Forty-four patients with PMS who met the inclusion criteria were included in the study. The mean age of the patients in the DN group was 44.6 ± 10 years, and in the exercise group, it was 41.4 ± 9.2 years. There was no statistically significant difference (p > 0.05) in age distribution between the groups. The DN group consisted of 14 female and eight male patients, and the exercise group comprised 20 female and two male patients. Females were significantly more in the exercise group (p < 0.05) in comparison to males. The mean BMI scores were 26.1 ± 2.1 kg/m2 and 25.1 ± 4.6 kg/m2 in the DN and exercise groups, respectively (p > 0.05). No significant differences (p = 0.364) were noted in BMI distribution between the study groups. The demographic characteristics of the groups are summarized in Table 1.

**Table 1:** Demographic characteristics of the study groups

		Dry needling group (n = 22)	Exercise group (n = 22)	P-value
Age (years) (mean ± SD)		44.6 ± 10	41.4 ± 9.2	0.266
Gender, n (%)	Female	14 (63.6)	20 (90.9)	0.034
	Male	8 (36.4)	2 (9.1)	
BMI (kg/cm²) (mean ± SD)		26.1 ± 2.1	25.1 ± 4.6	0.364
Occupation, n (%)	Unemployed	7 (31.8)	11 (50)	
	Desk worker	6 (27.2)	7 (31.8)	
	Heavy-lifting worker	-	1 (4.5)	
	Medical worker	4 (18.1)	2 (9)	
	Cyclist	2 (9)	-	
	Runner athlete	3 (13.6)	-	
	Driver	-	1 (4.5)	
Side of pain, n (%)	Right	10 (45.5)	13 (59.1)	0.273
	Left	12 (54.5)	9 (40.9)	

Although there was no significant difference between the groups in baseline scores of VAS, DN4, and LEFS (p > 0.05), the DN group's baseline ODI scores were significantly higher than those in the exercise group (p < 0.05). The within-group comparison had shown statistically significant differences from baseline scores with the post-treatment first-month and third-month scores in each group, in terms of pain, neuropathic pain, disability, and functional assessment values. The between-group comparison showed no significant difference in decreasing pain, reducing disability, and increasing functional status scores among the groups at post-treatment first-month and third-month assessments (Table 2).

**Table 2:** Comparison of VAS, ODI, LEFS, and DN4 values between study groups VAS: visual analog scale; ODI: Oswestry Disability Index; LEFS: Lower Extremity Functional Scale; DN4: Douleur Neuropathique 4.

		Dry needle group (n = 22)	Exercise group (n = 22)	P-value
VAS (mean ± SD)	Baseline	7.6 ± 1.6	7.8 ± 0.7	0.548
	1st month	3.4 ± 2.1	2.6 ± 1.7	0.183
	3rd month	2.5 ± 2.1	2.6 ± 1.1	0.929
ODI (mean ± SD)	Baseline	20.9 ± 8.5	32.6 ± 6.7	0.001
	1st month	10.2 ± 6.6	12.8 ± 7.2	0.230
	3rd month	7.4 ± 5.7	9.9 ± 7.7	0.226
LEFS (mean ± SD)	Baseline	41.7 ± 15.9	42.4 ± 15.8	0.880
	1st month	67 ± 12.2	67 ± 11.9	1
	3rd month	69.4 ± 12.9	71.3 ± 8.2	0.563
DN4 (mean ± SD)	Baseline	3.5 ± 2.1	4.1 ± 2.6	0.446
	1st month	1 ± 1.2	1.7 ± 1.6	0.115
	3rd month	0.9 ± 1.2	1.3 ± 1.4	0.331

## Discussion

In the present study, both a three-week exercise program and a total of three sessions of US-guided DN treatment once per week were found to be effective in pain intensity, neuropathic pain, and quality of life parameters in a three-month follow-up of patients with PMS. The ODI scores of the DN group were significantly higher at admission, which may suggest that patients with low quality of life due to pain are more often referred to outpatient pain clinics. In the literature, DN treatment of the piriformis muscle under US guidance is often in the form of case reports and there is no study comparing exercise programs and DN therapy efficacy. Our study will contribute to the literature because it evaluates both pain severity and the effect on lower extremity function, neuropathic pain, and quality of daily life.

PMS is a painful condition frequently associated with sciatic nerve symptoms caused by compression of the sciatic nerve due to anatomic variations in the piriformis muscle or secondary reasons [3,21]. Medical management and physical therapy are first-line options in PMS. Minimally invasive methods such as local anesthetics, steroids, botulinum toxin injections, and DN are all potentially effective therapies with few adverse effects [22]. Endoscopic release may be considered in patients for whom minimally invasive techniques are not effective [23]. In our study, no adverse effects were observed in any patients who underwent ultrasound-guided DN. An experienced physician and US-guided application minimize the risk of adverse effects.

In a study conducted by Tabatabaiee et al., 32 patients with PMS were randomly divided into two groups, patients who underwent three sessions of US-guided DN to the piriformis muscle and a control group. The primary outcome of the study was pain intensity (VAS) recorded at baseline and then 72 hours and one week after treatment. At the one-week follow-up, patients with PMS in the DN group showed clinically significant short-term improvement in pain intensity [1]. Similar to our study, DN treatment was performed under US guidance and a total of three sessions were performed once per week. In our study, the patients were divided into two groups, those who received an exercise program and those who received US-guided DN, and after the treatment, the first and third-month VAS, DN4, LEFS, and ODI scores were evaluated.

In a case series study, US-guided DN treatment was performed on the piriformis muscle and gluteus minimus, medius, and maximus muscles on three patients with the same symptom duration. All patients were administered eight to 12 sessions of DN for 10 days and were followed up for six months. Their symptoms and quality of life improved during this period and no adverse effects were reported [2]. We did not target other gluteal muscles in our study. We only targeted the piriformis muscle under US guidance. During the procedure, we advanced the needle through the gluteus maximus muscle to reach the piriformis muscle, which is located deep in the gluteus maximus muscle, but we did not perform needle therapy on the gluteus maximus muscle. In the literature, there are many injection techniques for the piriformis muscle using electromyographic, fluoroscopic, CT, or MRI guidance. However, US guidance offers several advantages, such as easy accessibility and portability, no radiation exposure, and direct imaging of neurovascular structures [24]. In our study, all DN treatment sessions were performed by the same experienced pain specialist by visualizing the needle insertion into the piriformis muscle under US guidance.

Stretching reduces muscle pain, prevents injury, and increases flexibility, thus it improves physical performance and ability [25]. Alarab and Unver reported that stretching exercises were effective in reducing pain and creating remarkable improvements in functional outcomes in patients with PMS [26]. In another study, various stretching methods were used to reduce piriformis thickness and enlarge medial rotation angles of the hip joints, and all methods were effective in decreasing symptoms [27]. Static stretching exercises are mostly preferred over other types of stretching methods in many musculoskeletal disorders [28,29]. In our study, we performed static piriformis stretching exercises for the exercise group and observed significant improvements in pain, disability, and functional assessment values, but there was no significant difference between the two study groups.

Piriformis syndrome is most common between the ages of 30 and 49 years and affects individuals of all physical activity levels and occupations. In our study, occupation diversity and the ranges of patients' ages were consistence with the literature [30].

Our study has certain strengths. The study's comprehensive assessment and management plan was clinically feasible, cost-effective, and patient-focused. After the treatment was completed, the patient's follow-up was three months. The limitations of the study can be listed as the relatively short follow-up period, the non-randomized study design, and the lack of a control group.

## Conclusions

Both treatment modalities were beneficial in reducing pain and disability and increasing the functional status of the patients with PMS in three months of follow-up. Stretching, on the other hand, is a simple, easy, and cheap conventional treatment approach that is accomplished without the requirement for supervision. In patients who cannot adapt to exercise programs, a single session of DN treatment to the piriformis muscle once per week under US guidance should be kept in mind as a minimally invasive treatment modality with no adverse effects.
